# ID 196 - Disseminação e Fortalecimento da Rede de Conhecimento dos Nats da Ebserh através da Série Temática sobre Avaliação de Tecnologias em Saúde Hospitalar

**DOI:** 10.5327/2237-9622.2025.v34s1.196

**Published:** 2025-11-25

**Authors:** Erika Maria Henriques Monteiro, Hérica Núbia Cardoso Cirilo, Luciana de Sousa Santos Costa, José Otávio do Amaral Corrêa

**Keywords:** avaliação de tecnologias em saúde, Nats, Ebserh, série temática, colaboração hospitalar, impacto social

## Abstract

**Introdução:**

A crescente complexidade tecnológica no ambiente hospitalar exigiu abordagens robustas para garantir que a incorporação de novas tecnologias fosse sustentável, ética e equitativa. Os Núcleos de Avaliação de Tecnologias em Saúde (Nats) desempenharam um papel essencial na produção de evidências que orientaram decisões de gestão hospitalar. A série temática sobre Avaliação de Tecnologias em Saúde Hospitalar (ATS-H), promovida pelo Nats do Hospital Universitário da Universidade Federal de Juíz de Fora (HU-UFJF) em parceria com a, representou uma iniciativa estratégica que não apenas disseminou conhecimento, mas também fortaleceu a colaboração entre os Nats da Rede da Empresa Brasileira de Serviços Hospitalares (Ebserh). Essa ação visou consolidar uma rede de compartilhamento de boas práticas e inovações, com impactos sociais diretos no acesso a tecnologias e no uso adequado delas, especialmente em contextos hospitalares públicos.

**Método:**

A série foi coordenada pelas editoras de seção dos Nats do HU-UFJF e do Hospital das Clínicas da Universidade Federal de Goiás (HC-UFG), com apoio da Ebserh Sede, e publicada na, um periódico de acesso aberto. Foram selecionados 11 artigos, que abordaram uma ampla gama de temas, desde revisões sistemáticas até relatos de experiências e análises de custo-efetividade. Os critérios de seleção incluíram relevância clínica, impacto social e potencial de aplicação na gestão hospitalar. A divulgação foi amplamente realizada pela Ebserh, assegurando que o conteúdo chegasse a diferentes Nats e gestores hospitalares.

**Resultados:**

A série temática ATS-H resultou na publicação de 11 artigos de grande relevância, tratando desde a eficácia da procalcitonina no manejo hospitalar até a elaboração de guias colaborativos entre Nats. O impacto social foi evidente, com a série promovendo a disseminação de evidências que apoiaram o uso racional e sustentável de tecnologias em hospitais universitários. O envolvimento de Nats de diversas regiões do Brasil ilustrou o valor da colaboração em rede, permitindo a adaptação de soluções conforme as necessidades locais e promovendo a equidade no acesso às inovações tecnológicas.

**Conclusão:**

A comparação dos achados dos artigos com outros estudos da literatura reafirmou o sucesso da série em promover o intercâmbio de conhecimento e a aplicação de evidências científicas na prática hospitalar. Além de fortalecer as capacidades institucionais dos Nats, a série ATS-H contribuiu significativamente para a coesão da Rede Ebserh, facilitando a criação de sinergias que ampliaram o impacto social da Avaliação de Tecnologias em Saúde. O fortalecimento dessa rede de colaboração não apenas promoveu a excelência na gestão de tecnologias, como também assegurou que o uso dessas inovações fosse equitativo, sustentável e centrado nas necessidades dos pacientes. Diante dos resultados obtidos, a continuidade dessa iniciativa se justificou como um caminho promissor para ampliar os impactos sociais e fortalecer a ATS no Brasil.

